# Elevated expression of p53 gain-of-function mutation R175H in endometrial cancer cells can increase the invasive phenotypes by activation of the EGFR/PI3K/AKT pathway

**DOI:** 10.1186/1476-4598-8-103

**Published:** 2009-11-16

**Authors:** Peixin Dong, Zhujie Xu, Nan Jia, Dajin Li, Youji Feng

**Affiliations:** 1 Hospital and Institute of Obstetrics and Gynecology, Fudan University Shanghai Medical College, Shanghai, PR China; 2 Department of Cardiovascular Medicine, Kanglian Hospital, Shanghai, PR China; 3 Department of Cardiology, Shanghai Jiao Tong University Affiliated First People's Hospital, Shanghai, PR China

## Abstract

**Background:**

p53 is the most commonly mutated tumor suppressor gene in human cancers. In addition to the loss of tumor suppression function and exertion of dominant-negative effects over the remaining wild-type protein, several p53 mutants can gain novel oncogenic functions (gain-of-function, GOF) that actively regulate cancer development and progression. In human endometrial cancer, p53 mutation is more often associated with aggressive nonendometrioid cancer. However, it was unknown if p53 mutants contributed to endometrial cancer progression through the GOF properties.

**Methods:**

To clarify the relationship between expression of p53 GOF mutation (p53-R175H) and invasive potential of human endometrial cancer KLE cells, we tested the consequences of up-regulation and down-regulation of p53-R175H in KLE cells by inducing p53-R175H expression vector or suppressing the p53 gene with short hairpin RNA.

**Results:**

We found that forced over-expression of p53-R175H significantly promoted cell migration and invasion, and induced activation of the epidermal growth factor receptor (EGFR)/phosphatidylinositol 3-kinase (PI3K)/AKT pathway. Conversely, suppression of p53-R175H with short hairpin RNA significantly inhibited cell migration and invasion, and resulted in attenuation of EGFR/PI3K/AKT pathway.

**Conclusion:**

These findings show for the first time that elevated expression of p53-R175H mutant may exert gain-of-function activity to activate the EGFR/PI3K/AKT pathway and thus may contribute to the invasive phenotype in endometrial cancer.

## Introduction

Endometrial cancer (EC) is the commonest gynecologic malignancy in the US and other Western nations [1]. Asian nations such as China and Japan have an incidence that is 4-5 times lower than in Western nations [2]. However, the incidence of EC in Asian countries has markedly increased in recent years [3]. Patients with advanced-stage EC frequently exhibit a poor prognosis, even after radical resection combined with radiotherapy or chemotherapy. These poor outcomes are closely associated with the progression and metastasis of the disease. Thus, a better understanding of the molecular mechanisms underlying the aggressive behavior of EC is necessary to identify potential targets for efficient therapy.

The tumor suppressor gene *TP53 *regulates the expression of genes involved in cell cycle arrest, apoptosis and DNA damage repair [4]. *TP53 *is mutated in more than half of human tumors. These mutations lead to single amino acid changes that influence the sequence-specific binding or the conformation of the mutant protein, abrogating its ability to induce the transcription of target genes (loss of function). It has been shown that p53 mutants exert dominant negative effects on co-expressed wild-type p53 (dominant-negative effects) [5,6]. Previous studies also indicated certain p53 mutations may confer oncogenic properties (gain-of-function, GOF) beyond their negative transdomination over the wild-type p53 tumor suppressor functions. These GOF effects include enhanced cancer cell proliferation and increased tumorigenicity *in vivo *[7-10], suggesting that GOF activity of p53 mutation may play an important role in tumor progression. However, little is known about GOF effects on tumor cell invasive activity. A common p53 mutant p53-R175H has been previously shown to possess a marked anti-apoptotic GOF in lung cancer cells [11]. In human EC, p53 mutations are more frequently identified in aggressive nonendometrioid cancer [12]. However, the precise role and the molecular mechanism of GOF properties of p53 mutants in EC progression and metastasis are poorly understood.

In this report, we sought to investigate the consequences of up-regulation and down-regulation of GOF p53 mutant (p53-R175H) on EC cell migration and invasion. Furthermore, we examined the molecular mechanisms by which p53-R175H over-expression lead to invasive phenotype in EC. We showed, for the first time, that elevated expression of p53-R175H in EC cells can display GOF effects to promote the invasive potential by activation of the EGFR/PI3K/AKT pathway.

## Materials and methods

### Cell lines and cell culture

The EC cell line KLE [13] harboring a p53 missense at codon 175 (p53-R175H, CGC > CAC) was obtained from the Cell Bank of the Chinese Academy of Sciences, Shanghai (China) and grown in Ham's F12 medium containing 10% heat-inactivated fetal bovine serum. The cells were maintained at 37°C under a humidified 5% CO_2 _atmosphere.

### Construction of expression vector expressing p53 GOF mutation p53-R175H and stable transfection

pCMV-p53 expression vector, which carries wt p53, was purchased from Clontech Laboratories, Inc. The corresponding empty vector named pCMV was made from pCMV-p53 by removing p53 sequence and religating the vector. pCMV-p53mt175 expression vector, which contains mutant p53-R175H, was generated from pCMV-p53 by the QuickChange mutagenesis system (Stratagene) following the manufacturer's recommendations. The primer sequence used for mutagenesis is 5'-GTGAGGCACTGCCCCCAC (forward) and 5'-GTGGGGGCAGTGCCTCAC-3' (reverse). The sequences of the mutation constructs were verified with an ABI 3100 sequencer (Applied Biosystems). Stable transfection of pCMV-p53mt175 and pCMV vector was performed using Lipofectamine PLUS Reagent (Invitrogen, Carlsbad, CA) according to the manufacturer's instructions. The transfected cells were selected in medium containing G418 (200 mg/ml). Independent clones were isolated, expanded, and screened for the expression of p53 protein expression by Western blotting. KLE cells transfected with pCMV-p53mt175 were designated R175H cells, and KLE cells transfected with pCMV vector were designated Vector cells.

### Knockdown of p53 GOF mutation p53-R175H using short hairpin RNA

The pSUPER-p53 RNAi System [14] was used to generate a stable KLE cell line with knocked-down p53 gene by an RNAi approach as described earlier [15]. In brief, KLE cells at 80% confluency were co-transfected with pSUPER-p53 and pPUR vector (BD Dickinson), or with pSUPER empty vector and pPUR vector, in the ratio 3:1 using the SuperfectR Transfection reagent (Qiagen), respectively. At 48 h after transfection, selection was started using 200 μg/ml G418 (Invitrogen). Resistant clones were characterized for levels of p53 protein by western blot. KLE cells transfected with pSUPER-p53 and pPUR were designated siRNA cells, and KLE cells transfected with pSUPER and pPUR were designated Control siRNA cells.

### In vitro Matrigel invasion assay

Matrigel invasion assay was performed using a 24-well invasion chamber system (BD Biosciences, Bedford, MA) with Matrigel membrane (8.0-μm pore), as described in our previous report [16]. Briefly, each 750 μl of Ham's F12 medium supplemented with 20% FBS and 10 μg/ml of bovine fibronectin (chemoattractant) were placed in the lower compartment of the chamber. In the prewarmed and rehydrated upper compartment, 2 × 10^4 ^cells in 500 μl of Ham's F12 medium supplemented with 20% FBS were added, and the cells were allowed to migrate through the intermediate membrane for 24 h at 37°C. Membranes were then fixed with 10% neutral-buffered formalin and stained in 5% Giemsa solution. The cells attached to the lower side of the membrane were counted in 10 high-powered (×200) fields under a microscope. Assays were done in triplicate for each experiment, and each experiment was repeated three times.

### In vitro cell migration assay

This migration assay was a modification of the assay described previously [17], which measured cell migration through an 8.0-μm pored membrane (BD Biosciences, Bedford, MA). In the lower chamber, 600 μl of Ham's F12 medium containing 20% FBS and 10 μg/ml of bovine fibronectin was placed. 2 × 10^4 ^cells in 100 μl of Ham's F12 medium supplemented with 20% FBS were placed in the upper chamber. After 6 h-incubation, the number of migrated cells (lower side of the membrane) was counted as described above. Assays were done in triplicate for each experiment, and each experiment was repeated three times.

### Western blot analysis

Whole cellular protein was obtained with M-Per Mammalian Protein Extraction Reagent (Pierce, Rockford, IL). The aliquots were separated on SDS-PAGE (10%) and transferred to nitrocellulose membranes. Antigen-antibody complexes were detected ECL blotting analysis system (Amersham Biosicences). The following primary antibodies were used: p53 (DO-1, Santa Cruz Biotechnology), EGFR antibody (2-18C9, Dako, Denmark), phosphorylated EGFR antibody (pEGFR, Tyr^1173^, Santa Cruz Biotechnology), AKT antibody (Cell Signaling Technology), phosphorylated AKT antibody (p-AKT, Ser^473^, Santa Cruz Biotechnology), ERK1/2 antibody (K-23, Santa Cruz Biotechnology), phosphorylated ERK1/2 antibody (p-ERK1/2, T183/Y185, Abcam plc) and G3PDH (sc-25778, Santa Cruz Biotechnology). EGFR inhibitor PD153035 was from Calbiochem.

### Statistical analysis

Statistical analyses were performed using the SPSS 10.0 software package (SPSS Inc., IL). The invasion and migration assay was analyzed by two-sided Student's *t *test. Statistically significance was defined as *P *< 0.05.

## Results

### Forced over-expression of p53-R175H by cDNA transfection promoted cell invasion and migration

**Figure 1:**
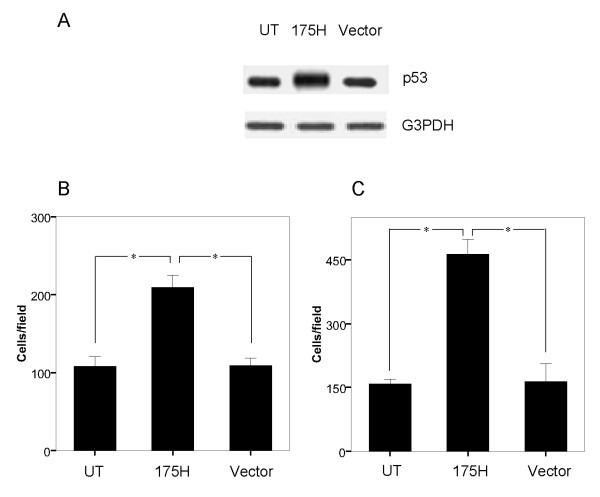
**Expression of p53-R175H is associated with enhanced invasion and migration in KLE cells**. (A) Western blot analysis showing p53 expression of p53-R175H in KLE cells after they had been transfected with a vector containing p53-R175H construct (R175H). Untransfected KLE cells (UT) and cells transfected with empty vector alone (Vector) served as the control. (B) Invasive ability of UT, R175H and Vector cells through the Matrigel-transwell membranes after 24 h of incubation. An asterisk indicates *P *< 0.05. UT and Vector cells served as the control. (C) Migratory property of UT, R175H and Vector cells through the transwell membranes after 6 h of incubation. An asterisk indicates *P *< 0.05. UT and Vector cells served as the control.

To evaluate whether p53-R175H expression associated with the invasive potential in EC, we chose human EC KLE cells to test the effects of forced over-expression of p53 GOF mutation R175H because this cell line expresses endogenous mutant p53-R175H. We transfected this cell line with either pCMV-p53mt175 or empty pCMV vector. The degree of p53-R175H protein expression was documented on Western blotting. As expected, Untransfected KLE cells (UT) and Vector cells expressed detectable amounts of p53-R175H, while R175H cells showed an increased p53-R175H expression (Figure 1A). We next compared the invasive activity of the cells in the Matrigel invasion assay. In contrast to UT and Vector cells, R175H cells showed a 2-fold increase in invasive migration through the Matrigel (*P *< 0.05) (Figure 1B). To evaluate the role of p53-R175H up-regulation on the motility properties of KLE cells, we also compared the cell motility using an in vitro cell migration assay. After 6 h-incubation, R175H cells were 3-fold more migratory than UT and Vector cells (*P *< 0.05) (Figure 1C). These results suggest that overexpression of p53-R175H can significantly promote the invasive and migratory function of KLE cells.

### Stable RNAi-mediated suppression of p53-R175H inhibited cell invasion and migration

**Figure 2:**
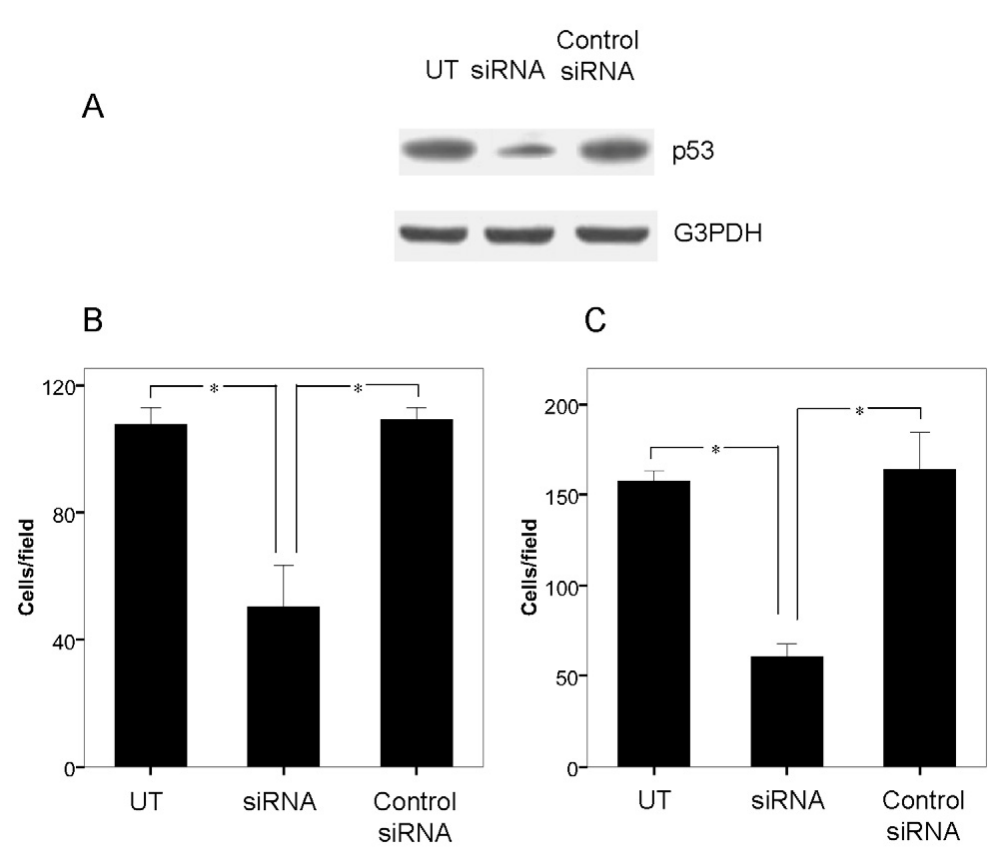
**Down-regulation of p53-R175H results in decreased cell invasion and migration**. (A) Expression of p53-R175H in KLE cells was inhibited by pSUPER-p53 vector (siRNA) as described in Materials and Methods. Cells were harvested and subjected to Western blotting to determine mutant p53 expression. Untransfected KLE cells (UT) and cells transfected with pSUPER empty vector (Control siRNA) served as the control. (B) Invasive ability of UT, siRNA and control siRNA cells through the Matrigel-transwell membranes after 24 hours of incubation. An asterisk indicates *P *< 0.05. UT and Control siRNA cells served as controls. (C) Migration potential of UT, siRNA and Control siRNA cells through the transwell membranes after 6 hours of incubation. An asterisk indicates *P *< 0.05. UT and Control siRNA cells were used as controls.

To further investigate the invasion-inhibitory effect of p53-R175H knockdown in KLE cells, we used RNAi approach to down-regulate the endogenous expression of p53-R175H in KLE cells. As shown in Figure 2A, UT and Control siRNA cells expressed detectable amounts of p53-R175H, while siRNA cells showed a decreased p53-R175H expression, as confirmed by Western blotting. We next tested the effects of p53-R175H down-regulation on cancer invasion and migration. Depletion of p53-R175H expression caused a significant decrease in cell invasion ability when compared to UT and Control siRNA cells (*P *< 0.05) (Figure 2B). Down-regulation of p53-R175H also significantly reduced KLE cell migration when compared to UT and Control siRNA cells (*P *< 0.05) (Figure 2C). These results suggest that repression of p53-R175H expression can lead to cell invasion and migration inhibition in KLE cells.

### p53-R175H induced activation of EGFR/PI3K/AKT pathway

**Figure 3:**
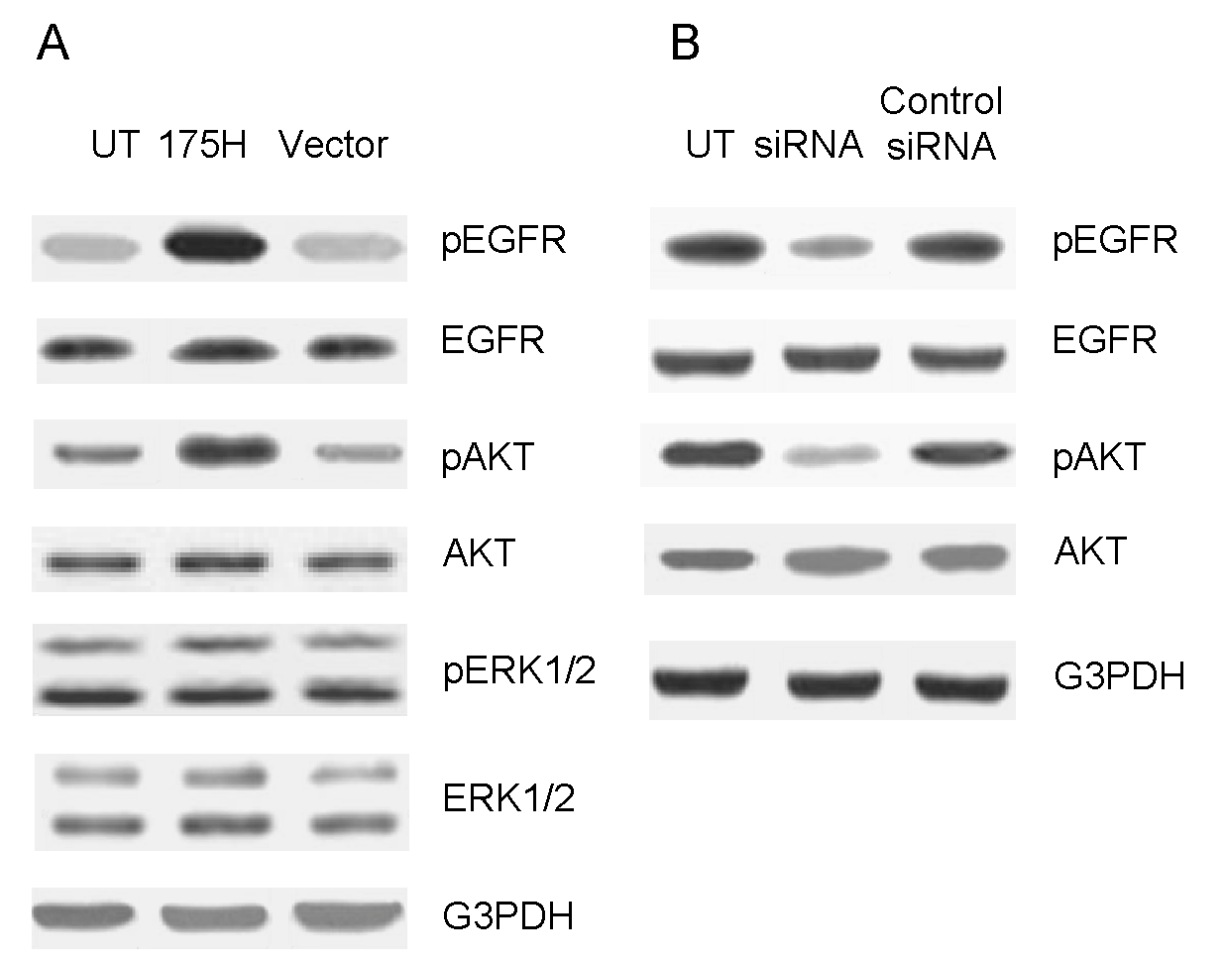
**p53-R175H induces activation of EGFR/PI3K/AKT pathway**. (A) Western blot analysis showing p53 expression in R175H, UT and Vector cells. UT and Vector cells served as the control. The membranes were stripped and probed with anti-pEGFR (pY^1173^), total EGFR, pAKT (ser^473^), total AKT antibody, pERK1/2, or total ERK1/2. The membranes were probed with anti-G3PDH antibody to assure even loading of proteins in each lane. (B) Expression of p53-R175H in KLE cells was inhibited by pSUPER-p53 vector (siRNA) as described in Materials and Methods. Cells were harvested and subjected to Western blot to determine mutant p53 expression. UT and Control siRNA cells served as the control. Membranes were stripped and probed with anti-pEGFR (pY^1173^), total EGFR, pAKT (ser^473^), or total AKT antibody. G3PDH was determined to ensure even loading of proteins in each lane.

Epidermal growth factor receptor (EGFR), a transmembrane phosphoglycoprotein, over-expressed in a variety of solid tumors, including EC [18], is the key molecular driver of oncogenesis and progression [19]. It has been shown that activated EGFR strongly correlate with tumor invasion and metastasis in EC [20]. Previous studies using osteosarcoma cells showed an association between EGFR and p53 mutations [21]. EGFR activation can lead to the activation of the downstream PI3K/AKT pathway [22,23]. Therefore, in this study, we investigated the effect of p53-R175H expression on EGFR/PI3K/AKT signal pathway using Western blotting after up-regulation or down-regulation of p53-R175H in KLE cells, respectively. When comparing with UT cells or Vector cells, R175H cells showed marked increase in constitutive activation of EGFR (p-EGFR, Tyr^1173^) and AKT (pAKT, Ser^473^) (Figure 3A). Conversely, the down-regulation of p53-R175H expression by RNAi resulted in inactivation of EGFR and AKT (Figure 3B). Because EGFR activation also results in the activation of the downstream MEK/ERK pathways, we next investigated the effect of p53-R175H expression on these pathways. Western blot analysis showed that no significant change in the levels of pERK1/2 was observed in R175H or Vector cells (Figure 3A). These findings suggest that p53-R175H-induced cell invasion and migration in KLE cells is dependent of EGFR/PI3K/AKT, but not EGFR/MEK/ERK signaling pathway.

### p53-R175H-induced AKT signaling is mediated by EGFR activation

**Figure 4:**
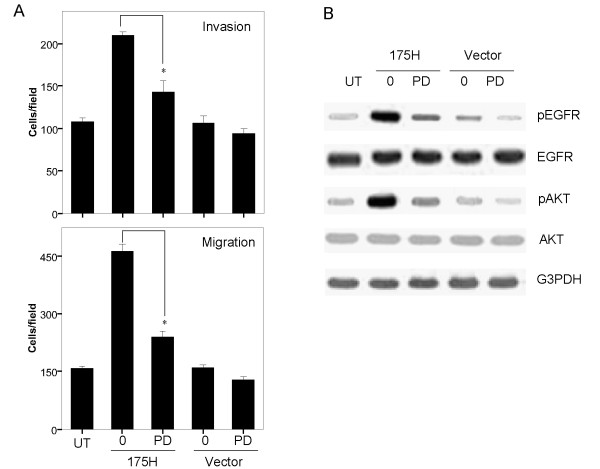
**p53-R175H-induced AKT signaling is mediated by EGFR activation**. (A) R175H, UT and Vector cells were seeded in 12-well plates and treated with PD153035 (20 μM). Cell invasion and migration assay were performed at 24 h and 6 h post treatment, respectively. An asterisk indicates *P *< 0.05. (B) R175H, UT and Vector cells were pretreated with PD153035 (20 μM) for 2 h and harvested for Western blot. Membranes were stripped and probed with anti-pEGFR (pY^1173^), total EGFR, pAKT (ser^473^), or total AKT antibody. G3PDH was determined to ensure even loading of proteins in each lane.

To further determine whether p53-R175H-induced increases in cell invasion and migration were EGFR-dependent, we performed the Matrigel invasion assay and migration assay in the presence of the EGFR inhibitor PD153035. At the concentration of 20 μM, pretreatment of KLE cells with EGFR inhibitor PD153035 caused significant reductions in cell invasion, and migration in R175H cells, respectively (Figure 4A and 4B). Although this treatment also decreased cell invasion and migration in Vector cells, the degree of inhibition was much less marked than that in R175H cells. Next, we sought to explore whether p53-R175H-induced PI3K/AKT signaling is mediated by EGFR activation, we pretreated KLE cells with specific EGFR tyrosine kinase inhibitor PD153035 (20 μM) for 2 h. Western blot analysis indicated that PD153035 significantly inhibited p53-R175H-induced EGFR and AKT activation (Figure. 4C). These results suggested that p53-R175H expression may promote the invasive function of EC cells, at least in part through the activation of EGFR/PI3K/AKT pathway.

## Discussion

Previous studies have demonstrated that gain-of-function of p53 cancer mutants could play important roles in carcinogenesis of various types of human cancers [24]. For example, p53 mutants can enhanced tumorigenic potential in nude mice and enhanced plating efficiency in agar cell culture [25]. These gain-of-functions of p53 cancer mutants acquire novel oncogenic activities, which may account for the close correlation between the expression of p53 mutants and the poor prognosis of patients with EC [26]. Moreover, p53 mutations are frequently detected in patients with high-grade serous and clear cell carcinomas, which are associated with an aggressive clinical course [12], suggesting the possibility that p53 mutations mediate features of the invasive and metastatic behavior of EC cells. However, little information is available regarding the consequence of GOF p53 mutant expression in EC progression. Therefore, in this study, we investigated the possible GOF roles of mutant p53 protein R175H in cell invasion and migration in EC KLE cells. Our data suggested that up-regulation of p53-R175H dramatically enhanced cell invasion and migration in KLE cells as shown in cell invasion and migration assay (Figure. 1). Conversely, down-regulation of p53-R175H by siRNA significantly attenuated the invasive potential of KLE cells (Figure. 2). Previous study showed p53-R175H can endow p53-negative T-ALL cells with the capacity to disseminate, and induce lymphohematopoietic metastatic potential and tissue invasiveness in SCID mice [27]. Thus, our results provided the in vitro evidence in support of the role of GOF activities of certain p53 mutant as an oncogene in EC progression.

Ample evidences support the critical role EGFR in tumor growth and progression, including angiogenesis, tumor cell proliferation and metastasis [19]. EGFR expression is increased in various cancer types, including endometrial cancer [18]. High levels of EGFR protein expression strongly correlates with tumor metastasis and poor prognosis in EC [28,29]. Previous study showed an association between p53 mutation and EGFR in bladder cancer [30]. Mutant p53-R175H has also been shown to activate the EGFR promoter in human osteosarcoma Saos-2 cells [21]. EGFR activation triggers the downstream PI3K/AKT and MEK/ERK signaling pathways [31]. Therefore, in this study, we determined the possible mechanisms by which p53-R175H could promote invasion ability in EC cells in regulation of EGFR/PI3K/AKT and EGFR/MEK/ERK pathway. Our present data suggested that over-expression of p53-R175H led to enhanced EGFR phosphorylation, and subsequently activation of PI3K/AKT pathway, but had no effect on the MEK/ERK pathway in KLE cells (Figure. 3). These results were supported by earlier reports that over-expression of EGFR in prostate and ovarian cancer was associated with activation of the PI3K/AKT signaling pathway [22,23]. Moreover, treatment of EGFR inhibitor abolished p53-R175H-induced cell invasion and migration and attenuated activation of AKT (Figure. 4), suggesting GOF effects of this mutant could drive invasive characteristics in EC cells through EGFR/PI3K/AKT-dependent pathway. However, our study is still limited for utilizing the cell line that expresses p53-R175H. Further experiments using p53-null EC cell lines are needed to confirm whether or not this mutation can show GOF properties to activate the EGFR/PI3K/AKT pathway, subsequently to enhance EC metastasis.

Overall, the results of this study show the possibility that GOF activity of p53-R175H contributes to an aggressive phenotype to EC cells, at least in part through the ability of p53-R175H to associate with active EGFR, subsequently resulting in the activation of its downstream PI3K/AKT signaling pathway.

## Competing interests

The authors declare that they have no competing interests.

## Authors' contributions

Dong PX designed research; Dong PX, Xu ZJ and Jia N carried out the molecular genetic studies; Dong PX, Li DJ and Feng YJ analyzed data; Dong PX wrote the paper. All authors read and approved the final manuscript.
